# Hunting for Answers: Assessing *Brucella* spp. Seroprevalence and Risks in Red Deer and Wild Boar in Central Portugal

**DOI:** 10.3390/pathogens13030242

**Published:** 2024-03-08

**Authors:** Humberto Pires, Luís Cardoso, Ana Patrícia Lopes, Maria da Conceição Fontes, Sérgio Santos-Silva, Manuela Matos, Cristina Pintado, Natália Roque, Leonardo Filipe Fonseca, Inês Morgado, Ana Sofia Dias, Luís Figueira, Ana Cristina Matos, João Rodrigo Mesquita, Ana Cláudia Coelho

**Affiliations:** 1 Polytechnic Institute of Castelo Branco, 6001-909 Castelo Branco, Portugal; humberto.s.pires@gmail.com (H.P.); cpintado@ipcb.pt (C.P.); nroque@ipcb.pt (N.R.); leonardo.g.fonseca@hotmail.com (L.F.F.); ines.morgadinha97@gmail.com (I.M.); sofiadias.biomed@gmail.com (A.S.D.); acmatos@ipcb.pt (A.C.M.); 2 Animal and Veterinary Research Centre (CECAV), Department of Veterinary Sciences, University of Trás-os-Montes e Alto Douro (UTAD), 5000-801 Vila Real, Portugal; aplopes@utad.pt (A.P.L.); mcfontes@utad.pt (M.d.C.F.); accoelho@utad.pt (A.C.C.); 3 Associate Laboratory for Animal and Veterinary Sciences (AL4AnimalS), 5000-801 Vila Real, Portugal; 4 School of Medicine and Biomedical Sciences (ICBAS), Porto University, 4050-313 Porto, Portugal; up202110051@edu.icbas.up.pt (S.S.-S.); jrmesquita@icbas.up.pt (J.R.M.); 5 Centre for the Research and Technology of Agro-Environmental and Biological Sciences (CITAB), University of Trás-os-Montes e Alto Douro (UTAD), 5000-801 Vila Real, Portugal; mmatos@utad.pt; 6 Research Center for Natural Resources, Environment and Society, Polytechnic Institute of Castelo Branco, 6001-909 Castelo Branco, Portugal; lmftfigueira@gmail.com; 7 Quality of Life in the Rural World (Q-RURAL), Polytechnic Institute of Castelo Branco, 6001-909 Castelo Branco, Portugal; 8 Epidemiology Research Unit (EPIUnit), Instituto de Saúde Pública da Universidade do Porto, 4050-600 Porto, Portugal; 9 Laboratory for Integrative and Translational Research in Population Health (ITR), 4050-600 Porto, Portugal

**Keywords:** *Brucella* spp., ELISA, Portugal, red deer, risk factors, wild boar

## Abstract

Between 2016 and 2023, a cross-sectional study was conducted in the central region of Portugal in order to better understand the epidemiology and public health risks resulting from the handling and consumption of game animals infected with *Brucella* spp. The seroprevalence and risk factors for *Brucella* spp. seropositivity were evaluated. Antibodies against *Brucella* spp. were determined using a commercial enzyme-linked immunosorbent assay (ELISA) according to the manufacturer’s instructions. Results showed that in the 650 serum samples collected from red deer (*n* = 298) and wild boars (*n* = 352) in Portugal, 21.7% (*n* = 141; 95% CI: 18.6–25.1%) tested positive. Wild boar had a significantly higher prevalence (35.5%; 95% CI: 30.5–40.8%) than red deer (5.4%, 95% CI: 3.1–8.6%; *p* ≤ 0.001). Risk factors for seropositivity were investigated using multivariable logistic regression models. The odds of being seropositive was 8.39 (95% CI: 4.75–14.84; *p* ≤ 0.001) times higher in wild boar than in red deer. Correlations between sex, age, body condition, and seropositivity could not be observed. The higher seroprevalence in wild boar suggests that this species may primarily contribute to the *Brucella* spp. ecology in central Portugal.

## 1. Introduction

Brucellosis is considered one of the most severe zoonoses globally [1,2]. This infectious disease is caused by bacteria of the genus *Brucella*, leading to abortion and infertility in various mammalian species. Species of the genus *Brucella* are Gram-negative intracellular facultative coccobacilli. In their native hosts, *Brucella* spp. are found in lymphoreticular tissue and primarily cause disease in reproductive tissues [3]. In the European Union (EU), brucellosis monitoring in ruminants is compulsory [4]. The European Centre for Disease Prevention and Control (ECDC) states that the surveillance systems for brucellosis have national coverage in all reporting EU/European Economic Area (EEA) countries, and the notification rate in the EU/EEA was 0.04 cases per 100,000 population in 2021 [5].

Humans contract the infection and disease through direct or indirect contact with infected animals or by consuming raw meat and unpasteurized dairy products. The primary transmission modes and pathways include contact with blood, body fluids, and aerosols through the digestive system, skin, mucous membranes, and respiratory tract [6]. The infection can cause mild to severe multiorgan illness and subclinical, acute, or chronic manifestations [6].

The *Brucella* genus has 13 recognized species, including *B. abortus*, *B. melitensis*, *B. suis*, *B. canis*, *B. ovis*, *B. neotomae*, the “marine” *B. pinnipedialis*, *B. ceti*, *B. inopinata*, *B. microti*, *B. pionis*, *B. vulpis*, and the recently described *B. pseudogrignonensis*. All these species are related, and the genus *Brucella* is linked to the genus *Ochrobactrum*, prompting the recommendation to rename them as *Ochrobactrum* [7].

The species most frequently connected to infections in humans is *B. melitensis*, followed by *B. abortus* and *B. suis*, and is the principal target of eradication efforts. *Brucella melitensis* is widely distributed worldwide and is associated with a considerable economic impact due to animal brucellosis [8].

Wild boars (*Sus scrofa*), domestic pigs, and various wildlife species have been identified as reservoirs of *B. suis* and *B. abortus* for both livestock and wildlife [8,9]. These species serve as natural hosts for *B. suis* biovar 2, which raises the possibility of zoonotic transmission to humans, other domestic animals, and domestic pigs, and can result in undiagnosed infections [10]. Wild boars are natural hosts for *B. suis* biovar 2, in which the infection passes inapparently, increasing the pathogen transmission risk to domestic pigs, other domestic animals, and humans [11]. Porcine brucellosis affects wild boars worldwide and is likely one of the most serious endemic diseases in Central European wild boar populations.

Wild boars are also known to be carriers of numerous other infectious diseases and zoonoses [12,13].

Brucellosis can be diagnosed through *Brucella* spp. isolation, DNA detection, or identifying specific antibodies. However, classical microbiological procedures, such as the isolation of *Brucella* spp., are rarely used in laboratories due to the required biosafety level 3, the health risk to laboratory personnel, and frequent failures to isolate bacteria. The Rose Bengal test (RBT), the complement fixation test (CFT), and the enzyme-linked immunosorbent assay (ELISA) are the most commonly used for screening purposes in wildlife [10,14,15]. As a more reliable test, ELISA has proven to be valuable for conducting epidemiological serosurveys [16].

Hunting is a more likely cause of *Brucella* spp. exposure in people than other occupational and leisure activities [17,18,19]. In Europe, the prevalence of *Brucella* spp. infection in wild boars has ranged from 0% to almost 60% [14,20,21,22,23].

Wildlife health is crucial for conservation, and in addition, wild animals are important sentinels for zoonotic pathogen surveillance [24,25].

The lack of natural predators, artificial feeding, and their high reproductive potential are the main causes of the wild boar population’s continuous growth in Europe, where it is estimated that there are up to 15 individuals per square kilometer [26].

The red deer (*Cervus elaphus*) is among the widely distributed ungulate species in Europe [27]. In Portugal, similar to trends observed across Europe, the population and range of this species have increased, which can be attributed to natural dispersion processes and reintroduction efforts [27]. Hunters’ exposure to wildlife while dressing game carcasses has been highlighted as a potential zoonotic danger [28]. The population of red deer has grown significantly throughout the Iberian Peninsula [29]. This discovery is most likely the result of field abandonment, forest advancement, and the introduction of hunting management practices [30,31]. Although related infections have been discovered in numerous places throughout the world, there have only been a few seroepidemiological studies on *Brucella* spp. in wild ungulates in Portugal [32,33]. The epidemiological state of wild animals in Portugal, such as red deer, is virtually unknown. This knowledge is critical for the implementation of prevention and control actions.

Studying the prevalence of brucellosis in wildlife is important since these animals are reservoirs for a number of agents known to impact public health. Additionally, as *Brucella* spp. heavily impact the reproductive capacities of infected animals, the abundance of these pathogens poses a commercial risk. As such, the occurrence of brucellosis in wild boar and deer serves as a marker of environmental contamination by these highly infectious and hazardous bacteria [34]. Therefore, this study aimed to determine the seroprevalence and risk variables related to *Brucella* spp. in wild boar and red deer in the Centre region of Portugal.

## 2. Materials and Methods

Sampling was conducted during the established hunting seasons (October to February) from 2016 to 2023. All animals were legally hunted by hunters for human consumption and were made accessible for post-mortem sampling. Serum samples were randomly obtained from free-roaming wild ungulates lawfully slain by hunters in the central region of Portugal, providing the opportunity to examine the presence of *Brucella* spp. antibodies. At each location, up to 10 animals were sampled in the first year, and this process was repeated with newly hunted animals each subsequent year. A total of 21 municipalities from the center of Portugal were sampled (Table 1 and Table 2). These areas have the highest concentration of wild ungulates in Portugal. A veterinarian performed a thorough examination of 650 wild ungulates from two unique species: 352 wild boar (*S. scrofa*) and 298 red deer (*C. elaphus*). Data on age, gender, bodily condition, and capture site were used to provide insight into the distribution of seropositive animals. The wild ungulates were separated into two age groups: juveniles and adults. Wild boars were considered juveniles until they reached the age of 8 months when they could become pregnant. Red deer were considered adults only when they were above 1.5 years old. Clinical signs were registered when an animal displayed one or more of the following: poor body or coat condition or macroscopic lesions in external or internal organs.

During the hunting season, which runs from October to February each year, blood samples were taken from the animals’ hearts or thoracic cavities. Blood was allowed to clot at room temperature before being brought to the laboratory. After centrifuging the blood samples at 1500× *g* for 10 min, the separated serum samples were stored at −20° C until further testing.

All sera were tested for the presence of anti-smooth-lipopolysaccharide (LPS) antibodies against *Brucella* spp. using a commercial ELISA kit (ID Screen^®^ Brucellosis Serum Indirect Multi-species ID.vet, Montpellier, France), according to the manufacturer’s recommendations, and the results were interpreted according to their guidelines. Briefly, specimens and controls were added to microwells diluted at 1/20. After the incubation and washing steps, a multi-species horseradish peroxidase (HRP) conjugate was added to the microwells fixing to the anti-*Brucella* antibodies, forming an antigen-antibody conjugate-HRP complex. Substrate and stop solutions were added and the resulting coloration measured to quantity specific antibodies present in the specimen.

The manufacturer’s negative and positive control samples were run in duplicate (first four wells). The optical densities (OD) of the tested samples and positive and negative controls were measured at 450 nm using an ELISA plate reader. The sample-to-positive-control OD ratio (S/P) was determined for each sample, with a cut-off value of 120 (S/P%).

The test was validated if the net mean value of the positive control OD (ODpc) was greater than 0.350: net ODpc > 0.350. The ratio of the net mean values of the positive and negative control ODs (ODpc and ODnc) was greater than 3. The formula used the absolute value of the net ODnc: net ODpc/|net Odnc| > 3. For each sample, the S/P percentage (S/P%) was calculated as follows:

$$\frac{S}{P}(\%)=\frac{{OD}_{sample}-{OD}_{NC}}{{OD}_{PC}-{OD}_{NC}}\times 100$$

### Statistical Analysis

To detect any risk variables linked with seropositivity, the outcome variable was dichotomized as seropositive versus seronegative. To determine whether there were significant differences between the groups, the Chi-square test was utilized. The odds ratio (OR) and 95% confidence interval (CI) of being seropositive in relation to the factors were modeled using multivariable logistic regression. Significant risk factors were then examined at *p* < 0.05 (two-tailed) using stepwise regression (Wald test value to enter *p* < 0.05). Backward elimination was followed by forward selection for each variable at a time, with 0.05 (two-tailed) as the significance level at each step. The fit of the models was assessed using the Hosmer and Lemeshow goodness-of-fit test [35]. The model was rerun until all remaining variables presented statistically significant values (*p* < 0.05).

All statistical analyses were performed using SPSS^®^ 25.0 software for Windows.

## 3. Results

In this study, the evaluation covered wild ungulates comprising 298 red deer, which accounted for 45.8% of the total, and 352 wild boars, making up 54.2%. In the present study, the overall seroprevalence was 21.7% (*n* = 141; 95% CI: 18.6–25.1%). Among the species that tested positive, wild boar had a significantly higher prevalence (35.5%; 95% CI: 30.5–40.8%) than red deer (5.4%, 95% CI: 3.1–8.6%; *p* ≤ 0.001) (Table 1).

Regarding municipal distribution, anti-*Brucella* antibodies were found in 19 (86.4%) out of the 22 municipalities studied (Table 2).

Serologic reactivity data according to species, sex, age, and clinical signs are presented in Table 3. The seroprevalence values among male and female animals were 22.9% (95% CI: 18.3–28.1%) and 20.6% (95% CI: 16.5–25.3%), respectively (Table 3). Regarding age, seroprevalence values were higher in juveniles (31.8%; 95% CI: 25.8–38.2%) than in adults (16.1%; 95% CI: 12.8–19.9%). Additionally, there were variations in seropositivity findings in relation to clinical signs. Seroprevalence was higher in animals with clinical signs (38.1%; 95% CI: 14.9–21.6%) compared to animals without clinical signs (38.1%; 95% CI: 29.4–47.5%) (Table 3).

In wild ungulates, three variables were associated (*p* < 0.05) with seropositivity. Seropositivity significantly correlated with species, age, and clinical signs. These variables were included in the multivariable logistic model. A backward stepwise conditional logistic regression was employed using all the statistically significant variables above. The multivariable logistic regression analysis of the odd ratio (OR) risk for being seropositive to potential risk factors is presented in Table 4. At the individual level, the odds of seropositivity to *Brucella* spp. were found to be higher for wild boar (OR = 8.4; 95% CI: 4.8–14.9) when compared to red deer (*p* ≤ 0.001).

## 4. Discussion

Every year, more than 500,000 new human brucellosis infections are anticipated to occur [36], with symptoms of the disease including intermittent fever, arthralgia, myalgia, abortion, exhaustion, and in certain cases, neurological abnormalities [37]. Humans can become infected by consuming contaminated animal products, inhaling infectious aerosols, or coming into contact with infected animals via conjunctiva or skin abrasions [38]. *Brucella* surveillance is critical worldwide to fill knowledge gaps about its transmission and reservoirs, especially given its zoonotic potential.

The present study found that both wild ungulate species were exposed to *Brucella* spp., with approximately 22% of the surveyed wild animals intended for human consumption having antibodies to *Brucella* spp. The reported seroprevalence of *Brucella* spp. by species ranged from 5.4% in red deer to 35.5% in wild boar, the most often hunted game animals in Portugal. Nevertheless, data on the presence of zoonotic diseases in wild ungulates used for human consumption are generally sparse in Portugal [12,13].

Brucellosis in small ruminants is still endemic in some areas of Portugal, representing a challenge to prevention and control [39,40,41,42,43,44,45]. *Brucella* spp. has also been detected in the Portuguese human population [46,47,48,49]. A previous study carried out in 332 wild boars reported an apparent seroprevalence of 26.5% in the North region of Portugal [32]. Several studies have been conducted in Europe to investigate the prevalence of *Brucella* infections in wild boar populations. In Italy, a survey conducted between 2001 and 2007 found a seroprevalence of 19.8% in 2267 wild boars, with all seropositive animals reacting to *B. suis* biovar 2 [50]. Subsequent studies in Italy [51] reported a 6.1% seroprevalence in 2015 and 13.5% in 2020, but the latter study did not conduct a verification of etiology. In 2021, *B. suis* biovar 2 was confirmed in Italian wild boars with a seroprevalence of 5.74%. In Belgium, a study conducted between 2003 and 2007 found an apparent seroprevalence of 54.9% in 1168 wild boars, confirming *B. suis* biovar 2 using culture and molecular typing [22].

In Poland, Ukraine, and the Netherlands, studies to determine the seroprevalence of *Brucella* infections in wild boar populations have also been conducted. In Poland, a seroprevalence of 24.5% was reported [52]. Ukraine reported seroprevalences ranging from 5% to 11.3% [53]. In the Netherlands, the occurrence of *Brucella* infections in wild boars ranged from 4.1% to 11.6% [54]. The differences in seroprevalence can be due to the sampling strategy, utilization of different serological methods, cut-off values, and sample types [55].

Limited research has been conducted on red deer. Anti-*Brucella* antibodies were found in red deer, chamois (*Rupicapra rupicapra*), and Alpine ibex (*Capra ibex*) in France [56]. Antibodies to *Brucella* spp. were not found in a sampled red deer population or other wild ruminants in Spain, indicating that red deer do not appear to be a suitable host for smooth *Brucella* spp. in the country [31,56,57,58]. Red deer were likewise shown to be *Brucella*-free in tests conducted in the Czech Republic [59] and in the different regions of Italy [50,51].

The higher prevalence in wild boar compared to red deer observed in the present study can be due to the presence of *B. suis*. Outside of the EU, feral pigs may harbor *B. suis* biovars 1 and 3, posing a risk of infection to both domestic pigs and humans [28]. It is possible that historical interactions between Iberian domestic pigs ranging freely and wild boars in the Iberian Peninsula may have promoted wild boar infection with *B. suis* biovar 2 [57,60]. Wild boars are thought to play an essential role as brucellosis reservoirs for farmed pigs even under natural environmental conditions [8,20,61]. Domestic pigs are epidemiologically connected to other major wild species, such as wild boars and ruminants, which serve as additional sources for the bacteria’s environmental expansion. Mammalian animals that interact with domestic ruminants in common habitats, especially wild ungulates, may promote the transmission of infectious agents such as *Brucella* spp. However, wild boars and domestic pigs continue to be the primary source of infection for ruminants residing in the same areas. Furthermore, the presence of small ruminants and wild animals near humans enhances the danger of disease transmission [10]. Concerning ruminants, while there is a suggestion that wild ruminants might carry brucellosis and potentially transmit it to domestic animals or humans, the prevailing belief is that these wild animals are more likely to be unintentional hosts of *Brucella* spp., acquiring the infection from infected livestock, rather than serving as a genuine reservoir of the disease for domestic animals [33].

The dynamics of wild boar and deer populations in the Iberian Peninsula are shifting, with a significant increase in Portugal. This change is caused by anthropogenic factors, such as urbanization, farming, deforestation, livestock expansion, loss of natural predators, and climate change, which have led to a closer proximity between livestock and domestic species and as a result, increased interaction between different species, thus facilitating the transmission of infectious diseases [62,63,64].

The lack of sex associations in relation to brucellosis’ apparent prevalence in wild boar was not unexpected, as comparable results have been obtained in another research [33]. Adult males are solitary and only interact with matriarchal groups during the mating season, whereas females dwell in matriarchal groupings. Adult wild boars had a higher apparent prevalence than juvenile wild boars, as expected, given their higher participation in reproduction [33].

Our results showed no difference between wild ungulates’ age and seropositivity for *Brucella* spp. This finding is not in line with previous studies, which found that prevalence increased dramatically with age [22,50]. This rise in older males and females was explained by longer and higher exposure to *B. suis* in older wild boars as well as evidence suggesting sexual activity in male wild boars above the age of two years [23].

According to our knowledge, this is the largest serological investigation on red deer in Portugal, and the largest seroprevalence study on wild boar in Portugal to date. Serological surveys are commonly used to investigate the presence and spread of infectious illnesses in wild animal populations. They are most commonly carried out as active monitoring and surveillance programs on blood samples obtained from hunted animals, comprising limitations in terms of the animal species, time of year, age category, and geographical distribution that can be evaluated [65]. The World Organization for Animal Health’s (WOAH) brucellosis diagnostic manual lists the various procedures for indirect pathogen identification and mentions the complement fixation test (CFT) and ELISA as standard methodologies [66]. Cross-reactivity with other bacteria, such as *Escherichia coli* O157, *Francisella tularensis*, *Moraxella phenylpyruvica, Yersinia enterocolitica* O:9, and some *Salmonella* serotypes, might result in false positive test findings [28]. Seroprevalence studies are valuable because they indicate exposure to *Brucella* spp. without specifying the inducing species. This approach is particularly relevant in wildlife, where the diversity of *Brucella* species makes it challenging to target specific antibodies. Additionally, seroprevalence studies can help assess the presence or spread of *Brucella* spp. within different wild species and classify them as exposed or non-exposed.

In recent decades, one of the most alarming trends for human and livestock health has been the emergence of zoonotic infectious disorders originating from wild animals. [67]. Wild ungulates are regarded as a reservoir of various infectious illnesses in the Iberian Peninsula, including tuberculosis, brucellosis, and paratuberculosis [68,69,70]. Wildlife interacts with humans, domestic cattle, and pet animals and so can serve as reservoirs and sources of infection and zoonotic diseases spread to the human interfaces. Wildlife’s potential function as a source of human zoonoses is an important public health issue [7,71,72].

*Brucella* spp. are largely thought to have originated in livestock and spread to wildlife [73]. Certain animal species can also keep the infection going in the absence of livestock interaction [74]. The establishment of huge areas under transfrontier conservation projects has encouraged the sharing of ecological systems by humans, wildlife, and domestic animals, potentially facilitating interspecies transmission of *Brucella* spp. [73,74]. Excreted material, such as vaginal excretions and abortion material from infected animals, is the primary source of contamination in feeding areas, pastures, and water as well as the primary cause of infection among animals [44].

The scarcity of data on brucellosis in red deer makes it challenging to assess the potential spread of this bacteria to wild boars, domestic pigs, other domestic animals such as ruminants, and also people.

Wildlife monitoring is required to detect changes in disease occurrence and assess the effectiveness of interventions. This monitoring in wild ungulates provides information for comparing distribution trends and prevalence in livestock, which can then be used to make disease control decisions in both types of populations and to assess the effects of any disease management action [75]. The impact of *Brucella* spp. is firstly on the reproductive system and related capacities in animals. These bacteria can heavily impact the farming industry as well as environmental protection programs. In humans, the disease usually has mild nonspecific symptoms, but it can have adverse consequences on working capacities, as it can cause sterility in men and miscarriages in women. In the case of highly virulent strains, it can cause blindness and neurological problems [3].

Game meat is not routinely tested for the presence of *Brucella* spp. during official meat inspections in Portugal and in many other countries in Europe. Until now, there has been no evidence of meat-borne transmission of *Brucella*, but this is a potential public health risk mainly associated with occupational exposure [76,77,78]. Occupational exposure to *Brucella* spp. poses a considerable risk to individuals in various professions, including hunters, veterinarians, farmers, or abattoir workers. They are at increased risk of contracting brucellosis due to their contact with wild animal’s blood, organs, and carcasses, some of which can be infected with *Brucella* spp. [28]. A systematic review and meta-analysis identified hunters as one of the groups most vulnerable to the occupational *Brucella* spp. infection, emphasizing the risk of direct or indirect contact with infected animals or their contaminated biological products. The main risk factors for hunters include exposure to aerosols and contact of non-intact skin with infected materials during activities such as field dressing and butchering of wild game [17]. Specific preventive measures, such as using personal protective equipment, thoroughly cooking meat, and practicing safe field dressing techniques, are crucial for reducing the risk of brucellosis among hunters [28].

### Study Limitations

The findings are based on a cross-sectional investigation with a convenience sample of wild ungulates in Portugal. The generalization of the results to the entire wild ungulate population within Portugal and beyond may be limited due to the sample size and geographic scope. This study relies on serological surveys for detecting antibodies to *Brucella* spp. and may have limitations in terms of sensitivity and specificity. While serological surveys are commonly used for active surveillance, they may not always accurately reflect the true infection status of animals. Complementary diagnostic methods, such as bacterial culture and molecular typing, could provide a more comprehensive understanding of *Brucella* infection in wild ungulate populations. At the same time, this study emphasizes the occupational risk of *Brucella* spp. exposure for hunters but does not provide detailed information on specific occupational risk factors or the effectiveness of existing preventive measures in the studied population. A more comprehensive assessment of occupational risk factors would enhance the practical implications of this study’s findings. While the present study provides valuable insights into the serological status of *Brucella* spp. in wild boar and red deer in Portugal, it is important to consider these limitations when interpreting and applying the findings to public health and wildlife management strategies.

The presence of *Brucella* spp. in household animals and wildlife increases public health risk, particularly for resource-limited communities living in this ecological environment [73,74]. However, controlling free-roaming wildlife is impractical; thus, controlling brucellosis in domestic animals may be critical to minimizing the danger to humans. Community participation and a “One Health” approach are also essential for disease prevention and control. Animal surveillance may need to be included with standard domestic animal surveillance because animals can be a direct source of human illness [79].

## 5. Conclusions

The current study emphasizes the necessity of a One Health, multidisciplinary strategy in assessing wild boar and red deer exposure to *Brucella* spp. and controlling brucellosis in the Centre region of Portugal. More investigation into the involvement of wildlife in the epidemiology of *Brucella* spp. infection is needed. By addressing wildlife reservoir concerns and integrating them into disease control strategies taken to safeguard the health of both animals and humans, stronger efforts can be made to protect animal and human health in the face of *Brucella* spp. abundance or circulation.

## Figures and Tables

**Table 1:** Seroprevalence of *Brucella* spp. infections in wild ungulates hunted for consumption in Portugal.

Wild Ungulates	No.	Prevalence (%) *p* < 0.001	CI 95% *
Wild boar (*Sus scrofa*)	125/352	35.5%	30.5–40.8%
Red deer (*Cervus elaphus*)	16/298	5.4%	3.1–8.6%
Total	141/650	21.7%	18.6–25.1%

* CI, 95% confidence interval.

**Table 2:** Seroprevalence of *Brucella* spp. by municipality.

Municipalities	No. Red Deer Pos./Total (%; CI *)	No. Wild Boar Pos./Total (%; CI *)	Positive Total (%; CI *)
Alcafozes	__	4/16 (25.0%; 7.3–52.4%)	4/16 (25.0%; 7.3–52.4%)
Castelo Branco	0/30 (0.0%; = 0.0–11.6%)	__	0/30 (0.0%; = 0.0–11.6%)
Cegonhas	__	1/8 (12.5%; 0.03–52.7%)	1/8 (12.5%; 0.03–52.7%)
Crato	2/12 (16.7%; 2.1–48.4%)	4/29 (13.8%; 3.4–31.7%)	6/41(14.6%; 5.6–29.2%)
Fratel	1/22 (4.6%; (0.01–22.8%))	3/13 (23.1%; 5.0–53.8%)	4/35 (11.4%; 3.2–26.7%)
Granja	0/10 (0.0%; 0.0–30.9%)	__	0/10 (0.0%; 0.0–30.9%)
Idanha-a-Nova	0/15 (0.0%; 0.0–21.8%)	5/11 (45.5%; 16.8–76.6%)	5/26 (19.2%; 6.6–39.4%)
Idanha-a-Velha	__	1/3 (33.3%; 0.0–90.6%)	1/3 (33.3%; 0.0–90.6%)
Lousa	__	23/44 (52.3%; 36.7–67.5%)	23/44 (52.3%; 36.7–67.5%)
Marvão	0/20 (0.0%; 0.0–16.8%)	3/11 (27.3%; 6.0–60.9%)	3/31 (9.7%; 2.0–25.8%)
Mata	1/13 (7.8%; 0.19–36.0%; 0.19–36.0%)	11/27 (40.7%; 22.4–61.2%)	12/40 (30.0%; 16.6–46.5%)
Monforte	0/10 (0.0%; 0.0–30.9%)	__	0/10 (0.0%; 0.0–30.9%)
Monte Fidalgo	3/60 (5.0%; 1.0–13.9%)	9/16 (56.3%; 29.8–80.3%)	12/76 (15.8%; 8.4–25.9%)
Niza	__	7/26 (26.9%; 11.6–47.8%)	7/26 (26.9%; 11.6–47.8%)
Ponte de Sor	__	8/25 (32.0%; 14.9–53.5%)	8/25 (32.0%; 14.9–53.5%)
Portalegre	__	20/49 (40.8%; 27.0–55.8)	20/49 (40.8%; 27.0–55.8)
Rosmaninhal	3/16 (18.8%; 4.1–45.7%)	10/23 (43.5%; 23.2–65.5%)	13/39 (33.3%; 19.1–50.2%)
Sarnadas do Ródão	1/32 (3.1%; 0.0–16.2%)	6/8 (75.0%; 34.9–96.8%)	7/40 (17.5%; 7.3–32.8%)
Tostão	__	1/9 (11.1%; 0.2–48.3)	1/9 (11.1%; 0.2–48.3%)
Vila Velha de Ródão	3/36 (8.3%; 1.8–22.5%)	7/28 (25.0%; 10.7–44.9%)	10/64 (15.6%; 7.8–26.9%)
Vale de Figueira	__	2/6 (33.3%; 4.3–77.8%)	2/6 (33.3%; 4.3–77.8%)
Vale Pousadas	2/22 (9.1%; 1.1–29.2%)	__	2/22 (9.1%; 1.1–29.2%)

* CI, 95% confidence interval; pos., positive.

**Table 3:** Screening for anti-*Brucella* antibodies in wild ungulates hunted for consumption in Central Portugal.

Variables	No. Red Deer Pos./Total (%; CI *)	No. Wild Boar Pos./Total (%; CI *)	Positive Total (%; CI *)
Sex	*p* = 0.782	*p* = 0.438	*p* = 0.480
Male	8/139 (5.8%; 2.5–11.0%)	61/162 (37.8%; 30.2–45.6%)	69/301 (22.9%; 18.3–28.1%)
Female	8/159 (5.0%; 2.2–9.7%)	64/190 (33.7%; 27.0–40.9%)	72/349 (20.6%; 16.5–25.3%)
Age	*p* = 0.509	*p* = 0.065	*p* ≤ 0.001 *
Juvenile	2/55 (3.6%; 0.4–12.5%	72/178 (40.5%; 3.2–48.1%)	74/233 (31.8%; 25.8–38.2%)
Adult	14/243 (5.8%; 3.2–9.5%)	53/174 (30.5%; 23.7–37.9%)	67/417 (16.1%; 12.8–19.9%)
Clinical signs	*p* = 0.266	*p* = 0.092	*p ≤* 0.001 *
Absence	16/287 (5.6%; 3.2–8.9%)	80/245 (32.7%; 26.8–38.9%)	96/532 (18.1%; 14.9–21.6%)
Presence	0/11 (0.0%; 0.0–28.5%)	45/107 (42.1%; 32.6–51.9%)	45/118 (38.1%; 29.4–47.5%)

* p < 0.05; pos., positive.

**Table 4:** Risk factors associated with *Brucella* spp. infection of wild ungulates in the Centre of Portugal.

Risk Factor	β ^a^	S.E. β ^b^	*p*	OR ^c^	95% CI ^d^ (OR)
Species	2.127	0.291	≤0.001		
Red deer				1	Reference
Wild boar				8.4	4.8–14.9

^a^ β: logistic regression coefficient; ^b^ S.E. β: standard error; ^c^ OR: odds ratio; ^d^ CI: confidence interval.

## Data Availability

The data presented in this study are available on request from the corresponding author.
